# A Dance and Yoga Intervention for Girls with Functional Abdominal Pain: Effects on Pain Frequency, Depressive Symptoms, Quality of Life, School Absenteeism, and Somatic Symptoms: A Randomized Controlled Trial

**DOI:** 10.3390/children13040542

**Published:** 2026-04-13

**Authors:** Sofie Högström, Anna Duberg, Anna Philipson, Ulrika L. Fagerberg, Stefan Särnblad

**Affiliations:** 1 University Health Care Research Center, Faculty of Medicine and Health, Örebro University, 701 82 Örebro, Sweden; anna.philipson@oru.se; 2 Department of Women’s and Children’s Health, Karolinska Institutet, 171 77 Stockholm, Sweden; ulrika.fagerberg@ki.se; 3 Center for Innovation, Research and Education, Västmanland Hospital Västerås, Region Västmanland, 721 89 Västerås, Sweden; 4 Department of Pediatrics, Faculty of Medicine and Health, Örebro University, 701 82 Örebro, Sweden; stefan.sarnblad@oru.se

**Keywords:** children, functional abdominal pain disorders, irritable bowel syndrome, randomized controlled trial, pediatric pain, disorders of gut–brain interaction

## Abstract

**Highlights:**

**What are the main findings?**

- The findings of this study suggest that a physical-activity-based intervention combining dance and yoga in a supportive group setting has the potential to reduce the frequency of abdominal pain post-intervention, with effects sustained 12 months post-baseline.

**What are the implications of the main findings?**

- In clinical practice, the implications suggest that the dance and yoga intervention may represent a valuable and accessible early treatment option for managing functional abdominal pain among young girls.
- The intervention provides children with practical body-based strategies that can support coping and self-management of pain and psychological distress. In addition to symptom reduction, the intervention may contribute to the development of regular physical activity habits early in life.

**Abstract:**

Background: Functional abdominal pain disorders (FAPDs) are common in youth and are often associated with depressive symptoms, school absenteeism, somatic symptoms, and low quality of life. This study aims to evaluate the effects of a dance and yoga intervention on abdominal pain frequency and associated symptoms over 24 months. Methods: This study presents analyses from a randomized controlled trial including 121 girls aged 9–13 years who were diagnosed with FAPDs. The intervention consisted of twice-weekly group sessions over eight months, combining dance and yoga. The primary outcome, maximum abdominal pain at 8 months, was published in 2022. Abdominal pain, depressive symptoms, health-related quality of life, school absenteeism, and somatic symptoms were prespecified as secondary outcomes in this study’s protocol. In the present manuscript, abdominal pain is analyzed as abdominal pain frequency. These secondary outcomes were assessed at 4, 8, 12, and 24 months. Both intention-to-treat and supportive per-protocol analyses were performed. Results: The intention-to-treat analysis showed a reduction in abdominal pain frequency in the intervention group compared with controls, with a mean difference of −1.10 with respect to the 95% CI (days per week) (−2.03 to −0.16; *p* = 0.02) at 8 months and −1.34 (−2.28 to −0.40; *p* = 0.005) at 12 months. No significant group differences were observed in the other outcomes. Per-protocol analyses showed similar or greater positive effects of the intervention. Conclusions: An intervention with combined dance and yoga has the potential to contribute to reductions in abdominal pain frequency at 8 and 12 months post-baseline in girls with FAPDs.

## 1. Introduction

Many school-aged children and adolescents suffer from abdominal pain and other problems associated with functional abdominal pain disorders (FAPDs) [1], such as anxiety [2,3,4], depressive disorders [2,4], somatization [3], school absenteeism [4,5], and low quality of life [2,4,6]. The global prevalence of FAPDs in children and adolescents is estimated at 11.7%, with a predominance among girls [7]. The pathophysiology of FAPDs is multifactorial, involving biological, psychological, and physiological components that interact in the microbiota–gut–brain axis, a bidirectional communication system between the gut and the brain [8]. Associated somatic symptoms and depression are identified as key predictors of persistent FAPDs. Notably, approximately 41% of children diagnosed with FAPDs continue to experience symptoms in early adulthood [9], and childhood recurrent abdominal pain is a strong predictor of severe mental illness later in life [10]. Girls with a diagnosis of pain are particularly at risk for developing mental health conditions [11].

Despite the significant burden of FAPDs, effective treatment options remain limited. There is a clear need for novel, effective, and accessible treatments [12] that address both the physical symptoms of FAPDs and associated psychological distress [3]. Dance is a dynamic cardiorespiratory physical activity that is popular among girls [13], and it has demonstrated both physical [14,15,16] and mental health benefits [16,17,18,19] for children and adolescents. Similarly, yoga-based practices have demonstrated potential to reduce FAPD symptoms [20,21] and depressive and anxiety symptoms among children [22]. Nevertheless, randomized controlled trials evaluating physically active interventions for this group are rare. Given this evidence, we hypothesized that an intervention combining dance and yoga in an undemanding setting with a focus on joy and playfulness, hereafter referred to as the Just-in-TIME (JiT) intervention, might be beneficial in reducing symptoms associated with FAPDs. The results from the primary outcome of this study have been reported previously and showed a significant reduction in abdominal pain intensity among girls who participated in the JiT intervention compared to controls [23]. The present study reports prespecified secondary outcomes and long-term follow-ups. Secondary outcomes were specified in this study’s protocol as follows: abdominal pain, depressive symptoms, health-related quality of life, school absenteeism, and somatic symptoms. In this study, abdominal pain was operationalized as abdominal pain frequency, reflecting a clinically important dimension of pain. Several of these outcomes have also been recommended for inclusion in core outcome sets for clinical trials in children with functional abdominal pain disorders [24]. The aim of this study was, therefore, to evaluate the effects of the 8-month JiT intervention on frequency of abdominal pain, depressive symptoms, health-related quality of life (HRQoL), school absenteeism, and somatic symptoms at 4, 8, 12, and 24 months post-baseline in girls aged 9–13 years with FAPDs.

## 2. Materials and Methods

### 2.1. Design

The JiT study was a prospective randomized controlled trial including 121 participants. The study is described in detail in a previously published study protocol [25] and was registered on ClinicalTrials.gov (ID: NCT02920268) and approved by the Regional Ethical Review Board in Uppsala, Sweden (Dnr 2016/082 1–2).

### 2.2. Recruitment and Study Sample

**Figure 1:**
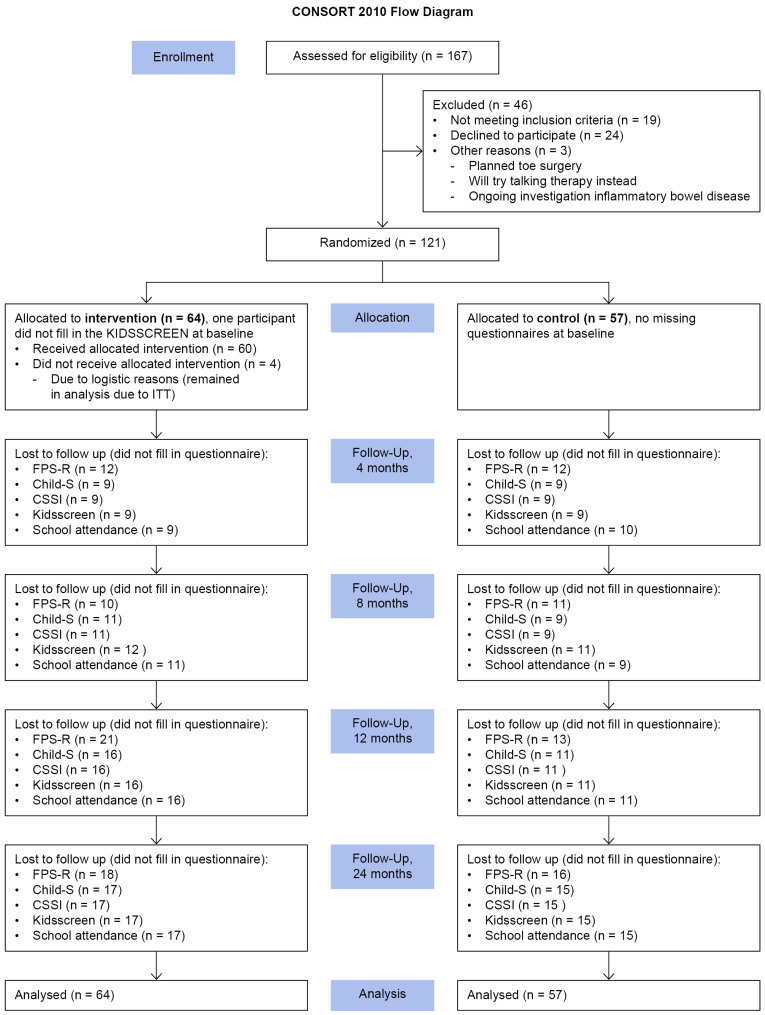
CONSORT flow diagram.

The inclusion criteria for the JiT study were girls aged 9–13 years diagnosed with irritable bowel syndrome (IBS) or functional abdominal pain (FAP) according to the ROME III criteria [26], with pain levels of 4 or higher at least once per week at baseline, as measured using the Faces Pain Scale—Revised (FPS-R) (scale 0–10) [27,28,29] and recorded in a pain diary. The exclusion criteria were co-existing celiac disease or inflammatory bowel disease, difficulty following oral instructions (hearing impairment, mental disability, or language difficulty), simultaneous treatment with cognitive behavioral therapy (CBT), and severe depression for which other treatment was needed. Recruitment of participants was carried out from the local registry of diagnoses at the Department of Pediatrics in Örebro and Västmanland County, Sweden, from primary and school health care in the region of Örebro and Västerås, Sweden, a counseling unit for children and adolescents in Örebro, Sweden, and through advertising in media and social media and on websites. After legal guardians provided written consent, the participants met with a pediatrician to verify the diagnosis and confirm the lack of exclusion criteria. If the participant had met a pediatrician in the previous six months, the diagnosis was verified according to medical records. The recruitment resulted in a total of 167 individuals, and the final sample included 121 participants who were randomly assigned, by an external statistician, to one of the two groups using minimization based on pain intensity and age at baseline [30] (intervention group n = 64; control group n = 57) (see flowchart (Figure 1)). The power calculation before the JiT study was based on the primary outcome of maximum abdominal pain and estimated 75 individuals per group to detect a clinically significant difference with 80% power (a = 0.05 two-tailed test), assuming a dropout rate of 20%.

### 2.3. The Just-in-TIME Intervention

The JiT intervention was carried out from September to May in Örebro (2016/2017), and in both Örebro and Västerås, Sweden, in 2017/2018 and 2018/2019. These are two mid-sized Swedish cities. In total, there were five JiT intervention groups with 7–14 participants, and in each city, two instructors who were specifically trained in the intervention each led one session per week. The JiT intervention was offered twice weekly for 8 months. Each session combined 30 min of dance practice; 25 min of yoga, including relaxation; and 5 min of brief reflection. The focus in all JiT sessions was on enjoyment, playfulness, and socialization—not on performance. The dance part contained several styles, such as show jazz and street dance, performed to popular music in various themes. Playful, guided improvisations were also included to encourage free movement. The yoga components consisted of calm, dynamic physical postures, breathing techniques, and focused attention. Postures were performed individually, in pairs, and as group exercises, often embedded in storytelling and always ending with guided relaxation and a brief massage (gentle pressure on forehead and shoulders). The instructors who were recruited for this study had previous experience in working with children and/or adolescents, and they had education in pedagogy or healthcare and in teaching dance and yoga. The instructors underwent a two-day course that included practical information about the standardized program, dance choreographies, yoga sequences, and essential elements of the intervention, such as teaching supportively and non-judgmentally. They received a written manual, and the course was followed by three booster sessions to ensure compliance with the method. All study participants had access to standard healthcare when needed. The participants in the control group were instructed to live as usual and were offered the intervention 24 months after baseline.

### 2.4. Measurements and Data Collection

Measurements were collected via questionnaires completed by the girls in a hospital auditorium at both study sites at baseline and at 4-, 8-, and 12-month post-baseline. Participants who were unable to attend the questionnaire sessions received the material by mail and returned it to the research team using prepaid envelopes. All participants were sent the evaluation material by mail 24 months post-baseline. Abdominal pain frequency was measured as the number of days with abdominal pain registered as 4 or higher on the FPS-R [27,28,29], recorded in a pain diary for one week. FPS-R is a widely used, validated self-reported measurement for pain in children [31] and has recently been evaluated in children with gut–brain interaction disorders, supporting its use in this population [27]. It consists of six faces representing increasing pain intensity, scored as 0, 2, 4, 6, 8, or 10. Depressive symptoms were measured with the Children’s Depression Screener (Child-S), an 8-question validated screening instrument for depressive symptoms developed for children aged 9–12 years. Each item is rated on a 4-point scale (0–3), resulting in a total score of 0–24, with higher scores indicating more depressive symptoms [32,33]. HRQoL was measured with KIDSCREEN-10, a well-established 10-question instrument with good internal consistency and test–retest reliability, developed to measure HRQoL in children aged 8–18 years [34,35]. Scores were transformed into T-scores according to the KIDSCREEN manual, where 50 represents the mean of the reference population and higher scores indicate better health-related quality of life [34]. School absenteeism was measured using two self-reported questions (“How often are you absent from school all day due to abdominal pain?” and “How often are you absent from school part of a day due to abdominal pain?”), with six response alternatives ranging from “never” to “several times each week.” Prior to analyses, the response alternatives were coded as follows: “never” = 0 days absent; “sometimes during each term” = 3 days absent; “once each month” = 5 days absent; “two to three times each month” = 10 days absent; “once each week” = 20 days absent; and “several times each week” = 40 days absent.

Absence from school for part of a day was estimated to be absence for a quarter of the day. Absences from school (for all or part of a day) were summed into a single summary score at each follow-up and converted to “days per semester.” Somatic symptoms were measured with the Children’s Somatic Symptoms Inventory (CSSI-24), a valid and reliable 24-item questionnaire assessing a variety of somatic symptoms [36,37]. Items were scored on a 5-point scale from 0 (not at all) to 4 (a lot) and converted into a summary score covering the past two weeks.

### 2.5. Statistical Analysis

A statistical analysis plan (SAP) was finalized after data access, data cleaning and outcome assessment, providing detailed operationalization and statistical specifications for the included outcomes. (SAP is available in the Supplementary Materials). Baseline characteristics of the study’s participants were summarized using descriptive statistics. Dependent variables were treated as continuous in the analysis and presented as mean and standard deviation or geometric mean and central variance in the descriptive statistics. Categorical data were presented with frequencies and percentages.

Statistical analyses were conducted using longitudinal analysis of covariance (ANCOVA) to assess differences between treatment groups over time, adjusting for baseline values and stratification variables (pain intensity at baseline and age). An unstructured covariance matrix was used to model correlations across repeated measures. Fixed effects included baseline values, age, and baseline pain intensity, along with their interactions with follow-up time: follow-up × study group, follow-up × age, and follow-up × baseline pain intensity. Strictly positive and right-skewed variables, such as school absenteeism and somatic symptoms (CSSI-24), were transformed with log-transformation prior to analyses in order to address skewness. Zero values were replaced by a pseudo-count of 0.5, treating a change from 0 to 1 and from 1 to 2 as equal in magnitude. No formal multiple-comparison adjustment was used, as the analysis was restricted to prespecified variables, and this study had limited power. Analyses were conducted according to intention to treat (ITT), including all randomized participants. Secondary analyses were conducted on the per-protocol (PP) population, excluding participants in the intervention group who attended less than 50% of the intervention sessions.

Manual instructions specific to the CSSI-24 [37] and KIDSCREEN [35] instruments were applied for managing missing items, applying the half-scale rule [38] using CHILD-S and pain diaries. Missing data at the score level were handled using multiple imputations via chained equations with a fully conditional specification. A total of 50 imputed datasets were generated, and results were pooled across imputations according to Rubin’s rules [39]. The imputation model included stratification variables, study group, and longitudinal outcomes, utilizing predictive mean matching for imputation. Missing data were assumed to be missing at random. See the flowchart for information about missing measurements for the dependent variables at each follow-up (Figure 1). For abdominal pain frequency, a post hoc sensitivity analysis was conducted within the ITT population among participants with available data at all five time points—using the same longitudinal ANCOVA model as used in the main analysis—to examine whether the results differed when the analysis was restricted to available cases rather than being based on the imputed dataset. The results were reported as estimated marginal means per group and time point, pooled across imputations, with raw standard deviations (SDs) from available cases. Group differences at follow-up were expressed as mean differences with 95% confidence intervals (CIs). For skewed outcomes (school absenteeism and somatic symptoms), log-transformed means and SDs were converted to geometric means and coefficients of variation, and between-group differences were reported as fold changes (exponentiated mean difference). Effect sizes were calculated as Cohen’s d, dividing the absolute mean difference between groups by the pooled baseline SD.

All tests were two-tailed with a 5% significance threshold. Statistical analyses and descriptives were performed using IBM SPSS Statistics version 25 (IBM Corp., Armonk, NY, USA).

## 3. Results

### 3.1. Descriptive Statistics

**Figure 2:**
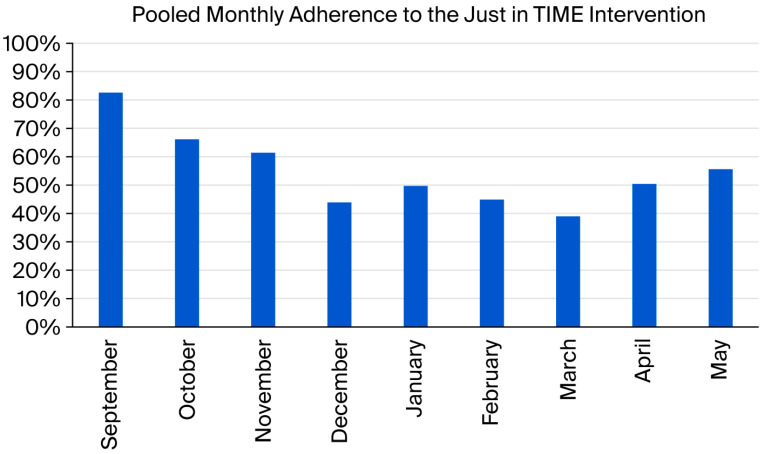
Pooled monthly adherence to the Just-in-TIME intervention across intervention periods.

On average, participants in the intervention group attended 55% of the offered JiT sessions during the 8-month intervention. Figure 2 provides information about monthly adherence pooled across all intervention periods.

Across outcomes, the proportion of participants with missing data at one or more time points was 39.7% for FPS-R (pain diaries), 30.6% for CSSI-24 and CHILD-S, and 31.4% for health-related quality of life and school absenteeism. The control group had 5–7 more missing observations per outcome than the intervention group.

The baseline characteristics of study participants are presented in Table 1.

### 3.2. Intention-to-Treat Analysis

ITT analysis showed a statistically significant reduction in abdominal pain frequency for the intervention group compared to controls at the 8- and 12-month follow-ups. The mean difference in the number of days per week with abdominal pain rated 4 or higher on the FPS-R was −1.10 (−2.03 to −0.16; *p* = 0.02) at 8 months and −1.34 (−2.28 to −0.40; *p* = 0.005) at 12 months, corresponding to medium effect sizes. The sensitivity post hoc analysis was conducted on 73 participants with pain diary data available at all five timepoints, including 39 in the intervention group and 34 in the control group. The results were consistent with the main analysis (Supplementary Table S1). There were no statistically significant differences in depressive symptoms, HRQoL, school absenteeism, or somatic symptoms in the ITT population (Table 2 and Table 3).

### 3.3. Per-Protocol Analysis

The PP analysis demonstrated a statistically significant reduction in abdominal pain frequency for the intervention group compared to controls across all four follow-up timepoints, with a mean difference of −1.09 (−2.04 to −0.13; *p* = 0.03) at 4 months, −1.68 (−2.66 to −0.71; *p* < 0.001) at 8 months, −1.65 (−2.63 to −0.67; *p* < 0.001) at 12 months, and −1.12 (−2.11 to −0.14; *p* = 0.02) at 24 months, corresponding to medium effect sizes. At the end of the intervention (8-month follow-up), depressive symptoms showed a significant decrease, with a mean difference of −1.84 (−3.67 to −0.01; *p* = 0.048) for the intervention group compared to controls and a small effect size. School absenteeism demonstrated a 0.62-fold change (95% CI 0.40–0.97), equating to a 38% reduction (−60% to −3% *p* = 0.04) in the intervention group compared to controls with a small effect size at the end of the intervention. Somatic symptoms showed a borderline significant difference at the end of the intervention, with a 0.74-fold change (95% CI 0.54–1.02) equating to a 26% reduction (−46% to 2%; *p* = 0.06) in the intervention group compared to controls and a small effect size. No other statistically significant differences were identified (Table 4 and Table 5).

## 4. Discussion

The JiT intervention, consisting of a combined dance- and yoga-based program delivered twice weekly over 8 months, significantly reduced abdominal pain frequency at 8 and 12 months in girls aged 9–13 years with functional abdominal pain disorders, as demonstrated in the intention-to-treat analyses. No effects were observed for depressive symptoms, health-related quality of life, school absenteeism, and somatic symptoms. Per-protocol analyses, including participants who attended at least half of the intervention sessions, showed significantly reduced abdominal pain frequency at all follow-ups, with sustained effects observed up to two years after treatment initiation. Reductions in depressive symptoms and school absenteeism were also observed at the 8-month follow-up. Additionally, a borderline significant improvement in somatic symptoms was observed at the end of treatment. No effects were found on quality of life.

The difference observed between the findings in ITT and PP analyses indicates that adherence could be associated with better outcomes, but this suggestion should be interpreted with caution. Nevertheless, the observed reductions in abdominal pain frequency, with a moderate effect as shown in the ITT analysis at both 8 and 12 months, may be an important finding with the potential to alleviate the daily burden for children and their families. This is supported by findings from a previous qualitative study conducted within this RCT, in which the analysis of interviews suggested that the JiT intervention was experienced as “A source of empowerment and well-being which facilitated personal growth and new ways of engaging in life” [40]. The effects on depressive symptoms and school absenteeism shown per protocol were small and not sustained over time, rendering their clinical relevance uncertain. Moreover, findings related to school absenteeism should be interpreted with caution due to methodological weaknesses in the measurement.

To our knowledge, no previous study has combined dance and yoga as an intervention for adolescents with FAPDs. However, other studies on shorter yoga interventions for adolescents with IBS, such as those by Kuttner et al. [41] and Evans et al. [42], which evaluated 4- and 6-week yoga interventions, have shown mixed effects with respect to gastrointestinal symptoms. This suggests that the 8-month JiT intervention, which combines yoga with dance and was specifically developed for the age group, may be more successful for this target group than a shorter yoga-only intervention. On the other hand, our findings partly align with those of Korterink et al. [43], who evaluated a 10-week yoga intervention for children with FAP and IBS, demonstrating effects on pain frequency, pain intensity, and school absenteeism but not on quality of life 12 months after intervention. No effects were found on HRQoL, which is in line with previous research suggesting that a reduction in IBS symptoms is not paralleled by improvements in the general quality of life of patients with IBS. Weerts et al. found that general quality of life is more likely to depend on concurrent psychological symptoms [44]. Since the participants in our study did not have high levels of depressive symptoms, this might explain the lack of effect on HRQoL.

While existing treatments, such as CBT and hypnotherapy, have demonstrated effectiveness in reducing pain and other symptoms in children with FAPDs [8,20,21], they may not be suitable for every child. Moreover, to our best knowledge, only gut-directed hypnotherapy has shown long-term effects (more than one year) on pain outcomes, somatization [20,45], anxiety, and depression [45]. The JiT intervention presents a promising complementary or alternative approach, particularly as it incorporates regular physical activity—a fundamental aspect that can be challenging for children with FAPDs, who often exhibit high levels of sedentary behavior and low physical activity [46]. This type of intervention also holds potential for reduced societal costs [47] and preventive health effects.

It is not possible to determine which specific components in the JiT intervention produced the observed effects; this study evaluated the whole intervention, combining dance- and yoga-based practice, including relaxation in a supportive group setting with no focus on performance. The effects might be explained by several positive physiological and psychological benefits of dance and yoga. Furthermore, the social aspect of the intervention might have given effects or constituted a basis for gaining effects. The undemanding setting for the JiT intervention, with a focus on enjoyment and the experience of the body in motion rather than on performance, may also have contributed to the observed effects. It is also possible that regular participation in the intervention reduced fear avoidance behavior and enhanced coping skills for these girls. Given the multifactorial pathogenesis of FAPDs, a strength of the JiT intervention is likely its ability to address physiological, psychological, and social aspects that may positively influence the gut–brain axis. From a broader societal perspective, these findings underscore the potential value of integrating non-pharmacological physical-activity-based group interventions into healthcare to address the multifaceted challenges faced by children with FAPDs.

An important consideration when interpreting the findings of this study is the relatively modest average adherence (55%). One possible explanation is the length of the intervention, which may have placed considerable demands on participating families, both logistically and in terms of sustained engagement over time. This may be particularly relevant in the present diagnostic group, where the nature of the symptoms can render participation more challenging. The findings of this study should be interpreted in light of several limitations. There was missing data for all outcomes at all follow-ups, which is often a challenge in clinical trials. The exact reasons for data loss could not be determined, as no formal analysis of the missing data was performed. Possible explanations might be missed assessments, practical issues, and/or parent–child coordination challenges. However, this was addressed using recommended imputation methods under the assumption of missing at random. Another methodological weakness is that it was impossible to blind participants to treatment. The number of participants did not reach the intended sample size, which means there was a potential risk of type II error, and the effects could have been missed or underestimated. Furthermore, as neither registry data nor psychometrically validated instruments regarding school absence were available, the questions used to measure school absenteeism were self-developed and not tested for psychometric validity. This introduces uncertainty into the data and necessitates caution when interpreting findings related to this variable. This study also has strengths, such as the randomized controlled study design and the longitudinal design that provides information about effects over time. Furthermore, the measurements for abdominal pain frequency, depressive symptoms, HRQoL, and somatic symptoms have good psychometric properties. The longitudinal analysis of covariance model allowed for baseline adjustments of the dependent variables.

## 5. Conclusions

This study suggests that a non-pharmacological dance and yoga intervention delivered in a supportive group setting may reduce abdominal pain frequency at 8- and 12-month follow-ups based on ITT analyses. The PP findings support these results, showing similar or greater effects. Future research should compare the JiT intervention with established treatments for FAPDs, such as CBT and hypnotherapy, and evaluate its effects across diverse FAPD populations.

## Figures and Tables

**Table 1:** Baseline characteristics of study participants.

	Intervention ITT ^a^ (n = 64)	Intervention PP ^b^ (n = 40)	Control (n = 57)
Age (years), mean (SD)	10.5 (1.4)	10.3 (1.3)	10.6 (1.3)
Diagnosis, n (%)			
IBS ^c^	27 (42.2)	16 (40)	20 (35.1)
FAP ^d^	37 (57.8)	24 (60)	37 (64.9)
Menarche, n (%)			
Yes	5 (8)	3 (7.5)	6 (10.5)
No	58 (92)	37 (92.5)	51 (89.5)
Abdominal pain frequency ^e^ (FPS-R ^f^), mean (SD)	4.4 (2.1)	4.4 (2.0)	4.0 (2.2)
Depressive symptoms (Child-S ^g^), mean (SD)	8.8 (4.3)	9.0 (4.4)	7.8 (4.5)
Quality of life (KIDSCREEN), mean (SD)	49.0 (7.0)	49.9 (7.0)	49.9 (7.1)
School absenteeism ^h^, geometric mean (CV)	3.5 (1.5)	4.0 (1.0)	4.1 (1.4)
Somatic symptoms, (CSSI-24 ^i^), geometric mean (CV)	14.1 (0.7)	14.4 (0.7)	12.0 (0.7)

^a^ Intention-to-treat. ^b^ Per-protocol; participants who attended at least 50% of intervention sessions. ^c^ Irritable bowel syndrome. ^d^ Functional abdominal pain. ^e^ Number of days with abdominal pain, rated 4 or higher on Faces Pain Scale–Revised, for one week. ^f^ Faces Pain Scale–Revised. ^g^ Children’s Depression Screener. ^h^ Number of days absent from school, due to abdominal pain, per semester. ^i^ Children’s Somatic Inventory-24.

**Table 2:** Treatment effect on abdominal pain frequency, depressive symptoms, and quality of life (ITT population).

Outcome/Follow-Up	Intervention (n = 64)	Control (n = 57)	Mean Difference (95% CI)	*p*-Value	Effect Size
Abdominal pain frequency ^a^ (FPS-R ^b^)					
4 months	2.9 (2.3)	3.5 (2.6)	−0.59 (−1.49, 0.32)	0.20	0.27
8 months	2.3 (2.3)	3.4 (2.7)	−1.10 (−2.03, −0.16)	0.02	0.51
12 months	2.2 (2.4)	3.5 (2.6)	−1.34 (−2.28, −0.40)	0.005	0.62
24 months	1.7 (2.3)	2.5 (2.7)	−0.82 (−1.75, 0.11)	0.08	0.38
Depressive symptoms (CHILD-S ^c^)					
4 months	7.8 (4.8)	8.6 (4.7)	−0.85 (−2.55, 0.85)	0.33	0.19
8 months	7.3 (5.2)	7.9 (4.2)	−0.56 (−2.28, 1.16)	0.52	0.13
12 months	6.3 (4.7)	7.5 (4.8)	−1.14 (−2.85, 0.57)	0.19	0.26
24 months	7.6 (5.3)	7.6 (4.5)	0.02 (−1.78, 1.82)	0.98	0.005
Health-related quality of life (KIDSCREEN)					
4 months	48.8 (8.3)	49.3 (7.7)	−0.47 (−3.64, 2.70)	0.77	0.07
8 months	50.4 (8.8)	49.9 (10.5)	0.54 (−2.80, 3.88)	0.75	0.08
12 months	51.9 (9.5)	50.6 (10.3)	1.31 (−1.97, 4.60)	0.43	0.24
24 months	50.7 (9.9)	49.2 (8.1)	1.50 (−1.70, 4.70)	0.36	0.21

Descriptive data are presented using estimated marginal means and standard deviations (SDs). Statistical analyses were conducted using longitudinal analysis of covariance (ANCOVA), adjusting for baseline values and stratification variables (baseline pain intensity and age). Missing data was handled using multiple imputation by chained equations (MICE). Effect sizes were calculated as Cohen’s d, dividing the absolute mean difference between groups by the pooled baseline SD. ^a^ Number of days with abdominal pain, rated 4 or higher on Faces Pain Scale–Revised, for one week. ^b^ Faces Pain Scale–Revised. ^c^ Children’s Depression Screener.

**Table 3:** Treatment effect on school absenteeism and somatic symptoms (ITT population).

Outcome/Follow-Up	Intervention (n = 64)	Control (n = 57)	Fold Change (95% CI)	*p*-Value	Effect Size
School absenteeism ^a^					
4 months	2.5 (1.5)	3.2 (1.8)	0.76 (0.50, 1.15)	0.19	0.26
8 months	2.1 (1.5)	3.1 (1.3)	0.69 (0.46, 1.05)	0.08	0.34
12 months	1.2 (1.6)	1.5 (2.2)	0.75 (0.48, 1.19)	0.22	0.27
24 months	1.0 (1.5)	1.2 (1.5)	0.79 (0.51, 1.24)	0.31	0.22
Somatic symptoms (CSSI-24 ^b^)					
4 months	10.8 (0.8)	12.2 (0.6)	0.88 (0.67, 1.16)	0.37	0.20
8 months	8.2 (1.0)	9.4 (1.0)	0.87 (0.66, 1.15)	0.33	0.22
12 months	8.8 (0.8)	10.0 (0.8)	0.88 (0.65, 1.18)	0.39	0.21
24 months	8.2 (1.2)	8.4 (0.9)	0.98 (0.69, 1.38)	0.90	0.04

Descriptive data are presented as geometric means with coefficients of variation (CV), calculated from estimated marginal means and standard deviations on log scale. Statistical analyses were conducted using longitudinal analysis of covariance (ANCOVA) on log-transformed variables, adjusting for log baseline values and stratification variables (baseline pain intensity and age). Missing data was handled using multiple imputation by chained equations (MICE). Effect sizes (Cohen’s d) were calculated on the log scale by dividing the absolute mean difference between groups by the pooled baseline SD. ^a^ Number of days absent from school, due to abdominal pain, per semester. ^b^ Children’s Somatic Symptoms Inventory-24.

**Table 4:** Treatment effect on abdominal pain frequency, depressive symptoms, and quality of life (PP population).

Outcome/Follow-Up	Intervention (n = 40)	Control (n = 57)	Mean Difference (95% CI)	*p*-Value	Effect Size
Abdominal pain frequency ^a^ (FPS-R ^b^)					
4 months	2.5 (2.3)	3.5 (2.6)	−1.09 (−2.04, −0.13)	0.03	0.50
8 months	1.7 (2.4)	3.4 (2.7)	−1.68 (−2.66, −0.71)	<0.001	0.77
12 months	1.9 (2.3)	3.5 (2.6)	−1.65 (−2.63, −0.67)	<0.001	0.75
24 months	1.5 (2.4)	2.6 (2.7)	−1.12 (−2.11, −0.14)	0.02	0.51
Depressive symptoms (CHILD-S ^c^)					
4 months	7.0 (4.8)	8.6 (4.7)	−1.58 (−3.39, 0.23)	0.09	0.36
8 months	6.1 (5.1)	7.9 (4.2)	−1.84 (−3.67, −0.01)	0.048	0.42
12 months	5.8 (5.0)	7.5 (4.8)	−1.68 (−3.51, 0.15)	0.07	0.38
24 months	7.5 (5.4)	7.6 (4.5)	0.06 (−1.84, 1.96)	0.95	0.01
Health-related quality of life (KIDSCREEN)					
4 months	50.0 (8.4)	49.6 (7.7)	0.45 (−2.44, 3.33)	0.76	0.06
8 months	52.1 (9.1)	50.1 (10.5)	2.01 (−2.29, 6.32)	0.36	0.29
12 months	53.4 (9.6)	50.9 (10.3)	2.49 (−1.19, 6.18)	0.18	0.35
24 months	51.1 (10.4)	49.5 (8.1)	1.68 (−2.04, 5.40)	0.38	0.24

Descriptive data are presented using estimated marginal means and standard deviations (SDs). The estimated marginal means for the control group differ between the intention-to-treat and per-protocol analyses due to model dependency and changes in the intervention group data. Statistical analyses were conducted using longitudinal analysis of covariance (ANCOVA), adjusting for baseline values and stratification variables (baseline pain intensity and age). Missing data was handled using multiple imputation by chained equations (MICE). Effect sizes were calculated as Cohen’s d, dividing the absolute mean difference between groups by the pooled baseline SD. ^a^ Number of days with abdominal pain, rated 4 or higher on Faces Pain Scale–Revised, for one week. ^b^ Faces Pain Scale–Revised. ^c^ Children’s Depression Screener.

**Table 5:** Treatment effect on school absenteeism and somatic symptoms (PP population).

Outcome/Follow-Up	Intervention (n = 40)	Control (n = 57)	Fold Change (95% CI)	*p*-Value	Effect Size
School absenteeism ^a^					
4 months	2.3 (1.4)	3.4 (1.8)	0.68 (0.43, 1.07)	0.10	0.40
8 months	2.0 (1.5)	3.2 (1.4)	0.62 (0.40, 0.97)	0.04	0.49
12 months	1.1 (1.6)	1.6 (2.2)	0.70 (0.43, 1.12)	0.14	0.37
24 months	0.9 (1.4)	1.3 (1.5)	0.68 (0.43, 1.10)	0.12	0.39
Somatic symptoms (CSSI-24 ^b^)					
4 months	10.1 (0.8)	12.3 (0.6)	0.82 (0.60, 1.11)	0.20	0.32
8 months	7.0 (1.0)	9.4 (1.0)	0.74 (0.54, 1.02)	0.06	0.47
12 months	7.8 (0.8)	10.0 (1.0)	0.78 (0.57, 1.06)	0.12	0.40
24 months	7.8 (1.1)	8.3 (1.2)	0.94 (0.67, 1.32)	0.72	0.10

Descriptive data are presented as geometric means with coefficients of variation (CV), calculated from estimated marginal means and standard deviations on log scale. Descriptive statistics for the control group differ between the intention-to-treat and per-protocol analyses due to model dependency and changes in the intervention group data. Statistical analyses were conducted using longitudinal analysis of covariance (ANCOVA) on log-transformed variables, adjusting for log baseline values and stratification variables (baseline pain intensity and age). Missing data was handled using multiple imputation by chained equations (MICE). Effect sizes (Cohen’s d) were calculated on the log scale by dividing the absolute mean difference between groups by the pooled baseline SD. ^a^ Number of days absent from school, due to abdominal pain, per semester. ^b^ Children’s Somatic Symptoms Inventory-24.

## Data Availability

The data presented in this study are available upon request from the corresponding author due to privacy and ethical restrictions.

## Supplementary Materials

The following supporting information can be downloaded at: https://www.mdpi.com/article/10.3390/children13040542/s1. The SAP is provided in the Supplementary Materials. The results from post hoc sensitivity analyses are provided in the Supplementary Materials (Table S1).

## Abbreviations

The following abbreviations are used in this manuscript:

ANCOVA	Analysis of Covariance
CBT	Cognitive Behavioral Therapy
Child-S	Children’s Depression Screener
CSSI-24	Children’s Somatic Symptoms Inventory-24
FAPDs	Functional Abdominal Pain Disorders
FPS-R	Faces Pain Scale—Revised
HRQoL	Health-Related Quality of Life
IBS	Irritable Bowel Syndrome
ITT	Intention to Treat
JiT	Just in TIME
PP	Per Protocol
