# Supplementary material for: A Dance and Yoga Intervention for Girls with Functional Abdominal Pain: Effects on Pain Frequency, Depressive Symptoms, Quality of Life, School Absenteeism, and Somatic Symptoms: A Randomized Controlled Trial
Source: Children (Basel). 2026 Apr 13;13(4):542. doi: 10.3390/children13040542 (PMC13114598; doi:10.3390/children13040542)
Supplement: Supplementary file 1 [file children-13-00542-s001.zip › Statistical Analysis Plan.pdf]

Statistical Analysis Plan (SAP)

Project: Just In TIME

Author: Henrik Imberg, Statistiska Konsultgruppen Sweden

Version and date: v1.0, 6 November 2024

Study overview

This study aims to analyse baseline and follow-up data collected across four visits per participant to assess differences between study groups on outcomes including depression (Child-S), pain frequency (measured with pain diary), health-related quality of life (KIDSCREEN), school absenteeism (measured with two questions), and somatic symptoms (CSSI-24). The analysis plan includes baseline adjustments and multiple imputation of missing data.

Statistical methods

Statistical analyses will be conducted using longitudinal analysis of covariance (ANCOVA) to assess differences between treatment groups over time, adjusting for baseline values and stratification variables. An unstructured covariance matrix will be used to model correlations across repeated measures. Fixed effects will include baseline values, age, and baseline pain intensity as covariates, with two-way interactions by visit (visit x study group, visit x age, and visit x baseline pain intensity). Strictly positive and right-skewed variables, such as school absenteeism and somatic symptoms (CSSI-24), will undergo log-transformation prior to analysis to address skewness. Zero values will be replaced by a pseudo-count of 0.5, treating a change from 0 to 1 and from 1 to 2 as equal in magnitude.

Missing data will be handled using multiple imputation by chained equations (MICE), with a fully conditional specification. A total of 50 imputed datasets will be generated, and results will be pooled across imputations according to Rubin's rules. The imputation model will include stratification variables, study group, and longitudinal outcomes, utilising predictive mean matching for imputation.

Results will be reported as estimated marginal means per group and timepoint, with raw standard deviations from available cases. Group differences by visit will be expressed as mean differences with 95% confidence intervals. For skewed outcomes (school absenteeism and somatic symptoms), log-transformed mean and standard deviation (SD) values will be converted to geometric means and coefficients of variation, with between-group differences presented as fold changes (exponentiated mean difference) or percentage changes (100 × (fold change − 1)).

All tests will be two-tailed with a 5% significance threshold. Statistical analyses will be performed using IBM SPSS Statistics (IBM Corp., Armonk, NY, USA).
