# Supplementary material for: A Dance and Yoga Intervention for Girls with Functional Abdominal Pain: Effects on Pain Frequency, Depressive Symptoms, Quality of Life, School Absenteeism, and Somatic Symptoms: A Randomized Controlled Trial
Source: Children (Basel). 2026 Apr 13;13(4):542. doi: 10.3390/children13040542 (PMC13114598; doi:10.3390/children13040542)
Supplement: Supplementary file 1 [file children-13-00542-s001.zip › Table.S1.pdf]

Table S1. Treatment effect on abdominal pain frequency, post-hoc sensitivity analysis on available cases (ITT population)

Outcome / Follow-up	Intervention (n = 39)	Control (n = 34)	Mean difference (95% CI)	p-value	Effect size
Abdominal pain frequency[a] (FPS-R[b])					
4 months	2.56 (2.3)	3.42 (2.6)	−0.87 (−1.88, 0.15)	0.09	0.40
8 months	1.92 (2.3)	3.15 (2.7)	−1.23 (−2.24, −0.22)	0.02	0.57
12 months	1.98 (2.4)	3.42 (2.6)	−1.45 (−2.46, −0.43)	0.005	0.67
24 months	1.65 (2.3)	2.46 (2.7)	−0.82 (−1.83, 0.20)	0.11	0.38

Descriptive data are presented using estimated marginal means and standard deviations (SDs).

Statistical analyses were conducted using longitudinal analysis of covariance (ANCOVA), adjusting for baseline values and stratification variables (baseline pain intensity and age) including participant with available data at all time points.

Effect sizes were calculated as Cohen's d, dividing the absolute mean difference between groups by the pooled baseline SD.

[a]Number of days with abdominal pain, rated 4 or higher on Faces Pain Scale–Revised, for one week

[b]Faces Pain Scale–Revised
